# Determination of Food Oxalates Using Silica–Titania Xerogel Modified with Eriochrome Cyanine R

**DOI:** 10.3390/s18030864

**Published:** 2018-03-15

**Authors:** Maria A. Morosanova, Zahar V. Samodelov, Elena I. Morosanova

**Affiliations:** Analytical Chemistry Division, Chemistry Department, Lomonosov Moscow State University, Moscow 119234, Russia; m.a.morosanova@gmail.com (M.A.M.); conc.lab.student@gmail.com (Z.V.S.)

**Keywords:** oxalate determination, silica–titania xerogel, triphenylmethane dyes, food oxalates, solid phase spectrophotometry

## Abstract

The interaction of silica–titania xerogel with triphenylmethane dyes (pyrocatechol violet, chrome azurol S, eriochrome cyanine R) has been investigated to create a new sensor material for solid phase spectrophotometric determination of food oxalates. The complex forming reaction between xerogel incorporated titanium(IV) and triphenylmethane dyes has been studied; half-reaction periods, complex composition, equilibrium constants, and xerogel sorption capacity have been calculated for each dye. Eriochrome cyanine R (ECR) is characterized by the shortest half-reaction period, the smallest equilibrium constant, and the greatest capacity; it has been chosen for the sensor material construction because titanium(IV)-ECR complex is formed faster and can be destroyed easier than other studied complexes. The interaction of this sensor material with oxalates has been described: the presence of oxalates causes sensor material discoloration and the absorbance is used as analytical signal. The analytical range is 35–900 mg/L (LOD 10.5 mg/L, *n* = 7). High concentrations of interfering inorganic anions, organic acids, and sucrose did not affect oxalate determination. Proposed solid phase spectrophotometric procedure has been successfully applied for the determination of oxalates in food samples (sorrel, spinach, parsley, ginger, and black pepper) and the results are in good agreement with HPLC oxalate determination.

## 1. Introduction

Oxalates can be an important health risk, because consumption of large amounts of oxalate causes secondary hyperoxaluria, which often leads to kidney stone formation [1,2]. An excessive oxalate intake can be responsible for acute renal failure and it also reduces the bioavailability of calcium and magnesium [3]. The main sources of dietary oxalate are high-oxalate containing plants [1]. Given the risks of oxalate overconsumption, oxalates are routinely monitored in food quality control.

Separation methods [2,4,5,6,7,8] and methods employing oxalate oxidase enzyme [9,10,11,12,13,14,15,16,17,18,19] are the most widely used for oxalate determination. Classical methods for oxalate determination include capillary electrophoresis [2] and chromatographic methods [4,5,6]. Modern development of these methods is focused on the improvement of sensitivity and selectivity. This can be achieved through using oxalate oxidase enzyme reactor [7] or adding chemiluminescence causing reagent [8] after the chromatographic separation. However, these methods still require complex equipment and are performed in the professional laboratories, while the importance of food analysis dictates the need for simpler routine methods that can be used on-site.

Enzymatic methods and biosensors using immobilized oxalate oxidase provide several approaches for sensitive and simple oxalate determination procedures, making them available for widespread application [9,10,11,12,13,14,15,16,17,18,19]. One of the approaches is using flow injection technique with amperometric [9] or spectrophotometric [10] detection of the hydrogen peroxide provided by oxalate oxidase reaction. Another approach using oxalate oxidase is the construction of amperometric biosensors [11,12,13,14,15,16,18,19]. These biosensors are based on monitoring of hydrogen peroxide oxidation current at very high potential values which causes the problem of the oxidation of other interfering substances [12]. This interference problem is overcome by adding peroxidase enzyme or mediator substances to enhance the signal of hydrogen peroxide [11,12]; however, ascorbic acid and metal ions still have a significant impact on the enzyme used in these biosensors. Another disadvantage of the enzyme based methods is their storage time: the half-time of a biosensor is about one month.

Alternative approach to simple and reliable analysis is chemically active sensor materials. Such materials for the determination of anions, including oxalates, can be constructed using the ability of anions to form stable complexes with metal ions. As oxalate complexes are not colored, spectroscopic oxalate determination is based on decomposition of colored or fluorescent metal complexes with different substances, in solution [20,21] or in solid phase [22,23]. In [22] and [23], metal ions interact with oxalate in solution and then the excess of metal ions interact with solid phase (silica-gel modified with complexing reagent [22] or carbon dots [23]). These sensor materials cannot be used without adding a metal ion solution. These sensor materials have been applied for oxalate determination in aqueous solutions and urine [22,23].

Sol–gel silica–titania materials are stable and versatile in textural properties due to the nature of sol–gel synthesis, and the titanium(IV) retains its complexing ability which can be used to create sensor materials [24]. Colored titanium(IV) complexes that are embedded in the xerogel matrix can also be decomposed by anions: we have used silica–titania xerogels modified with phenolic compounds and hydrogen peroxide for solid phase spectrophotometric fluoride determination [25]. Titanium(IV) complexes with triphenylmethane dyes in solutions are well studied [26] and can be used for anions determination.

The aim of the present work was to synthesize silica–titania xerogel modified with triphenylmethane dyes and apply it as a sensor material for solid phase spectrophotometric determination of oxalates in food.

## 2. Materials and Methods

### 2.1. Reagents and Apparatus

The following reagents were purchased from Acros Organics: triphenylmethane dyes (TPMD): pyrocatechol violet (PV), eriochrome cyanine R (ECR), chrome azurol S (CAS), hydrochloric acid, titanium(IV) tetraethoxyde, tetraethyl orthosilicate, and oxalic acid. All the reagents were of analytical grade, titanium(IV) tetraethoxyde was of technical grade. Stock solutions of oxalic acid and TPMD were prepared with doubly distilled water. Only freshly prepared solutions were used.

Silica–titania xerogels were obtained by drying in Ethos microwave equipment (Milestone, Sorisole, Italy). Surface area, porosity BET analysis, and BJH pore distribution analysis were carried out with ASAP 2000 (Micromeritics, Norcross, GA, USA). Absorbance of solutions (l = 1.0 cm) and xerogels water suspensions (l = 0.1 cm) was measured using Lambda 35 spectrophotometer (PerkinElmer, Waltham, MA, USA) equipped with 50 mm integrating sphere (Labsphere, North Sutton, NH, USA). The pH value was measured using Expert-001 (Econix Expert, Moscow, Russia) potentiometer with pH sensitive electrode. Chromatographic determination was conducted using MAESTRO HPLC system (Interlab, Moscow, Russia). A Kromasil C18 column (250 × 4.6 mm, 5 μm particle size) served as the stationary phase, mobile phase was distilled water (pH 2). UV detection was performed at 220 nm and a flow rate of 1.0 mL·min^−1^.

### 2.2. Synthesis of Silica–Titania Xerogel

Silica–titania xerogel was obtained using earlier developed procedure [25]: 20.0 mL of 0.05 M hydrochloric acid in 50% ethanol solution was added to 10.0 mL of the precursors mixture (12.5% titanium(IV) tetraethoxyde, 87.5% tetraethoxysilane) while stirring. The wet gel was formed in the next 72 h. The wet gel was dried at 800 W microwave irradiation for 10 min to get dry xerogel. Obtained xerogel powder was ground and sorted into fractions with different particle sizes using a set of sieves. The fraction of 0.10–0.16 mm sized xerogel particles was used.

### 2.3. General Procedure for the TPMD—Silica–Titania Xerogel Interaction Study

0.10 g of silica–titania xerogel was added to 5.0 mL of solution, containing 2.0 × 10^−5^ M to 1.0 × 10^−3^ M of TPMD at different pH and the obtained mixture was shaken (2–30 min). Then the xerogel absorbance spectrum was recorded. The optimal conditions were chosen in order to reach the maximal absorbance.

### 2.4. Preparation of the Sensor Material—Eriochrome Cyanine R Modified Silica–Titania Xerogel (Si-Ti/ECR)

10.0 g of silica–titania xerogel was added to 90.0 mL of the 5.0 × 10^−4^ M eriochrome cyanine R solution at pH 2.0. After shaking for 30 min, modified silica–titania xerogel (Si-Ti/ECR) was washed three times with distilled water and dried at room temperature overnight.

### 2.5. General Procedure for the Oxalate—Si-Ti/ECR Interaction Study

0.10 g of Si-Ti/ECR was added to 5.0 mL of solution, containing 200 mg/L oxalate at different pH and the obtained mixture was shaken (2–20 min). Then Si-Ti/ECR absorbance was measured at 570 nm. The optimal conditions were chosen in order to reach the maximal absorbance loss in presence of oxalate.

Calibration curve was constructed using the absorbance of Si-Ti/ECR after interaction with the standard solutions of oxalate in the range of 10 mg/L–2000 mg/L in optimal conditions (pH 3.0, λ 570 nm, time of contact 15 min) in triplicate. Least squares method was used to obtain the calibration curve. The limit of detection (LOD) was calculated as 3∙standard deviation of the blank absorbance (*n* = 7) divided by the slope value. The limit of quantitation (LOQ) was calculated as 10∙standard deviation of the blank absorbance (*n* = 7) divided by the slope value.

For each interfering compound a set of solutions with 200 mg/L oxalate concentration and various concentrations of the interfering compound was prepared. 0.10 g of Si-Ti/ECR was added to 5.0 mL of each of those solutions (pH 3.0, time of contact 15 min) and Si-Ti/ECR absorbance was measured at 570 nm. The interference threshold is defined as the concentration of interfering compound causing 10% change in the oxalate concentration calculated using the calibration curve, while all lower concentrations of interfering compound cause less than 10% change.

### 2.6. Sample Preparation and Solid Phase Spectrophotometric Determination Procedure

10.0 g of food sample was homogenized in 100.0 mL of distilled water, boiled for 15 min. After cooling the solution was filtered. The solution pH was adjusted to 3.0 and finally solution was diluted to the mark of 100.0 mL. Then sample solution was diluted five times.

0.10 g of Si-Ti/ECR was added to 5.0 mL of the treated sample solution, the obtained mixture was shaken for 15 min, and then Si-Ti/ECR absorbance was measured at 570 nm. The concentration of soluble oxalate in the sample was calculated using the method of standard addition or using the calibration curve.

## 3. Results

### 3.1. Interaction of Silica–Titania Xerogels with Triphenylmethane Dyes

In our previous works the interaction of silica–titania xerogels with hydrogen peroxide [27], salicylic acid derivatives [28], and phenolic compounds [29,30] has been studied. This interaction leads to the formation of colored Ti(IV)-analyte complex, similar to the complexes formed in the solution. In the present work we studied the interaction of silica–titania xerogels with triphenylmethane dyes (TPMD): eriochrome cyanine R (ECR), pyrocatechol violet (PV), and chrome azurol S (CAS). The interaction of TPMD (ligand, L) with silica–titania xerogel incorporated titanium(IV) is described by the equation

(1) $$\equiv \bar{\mathrm{Ti}-{\left(\mathrm{OH}\right)}_{n}}\;+\;\mathrm{nHL}\;=\;\equiv \bar{\mathrm{Ti}-L_{n}}{\;+\;\mathrm{nH}}_{2}O.\;$$

**Figure 1:**
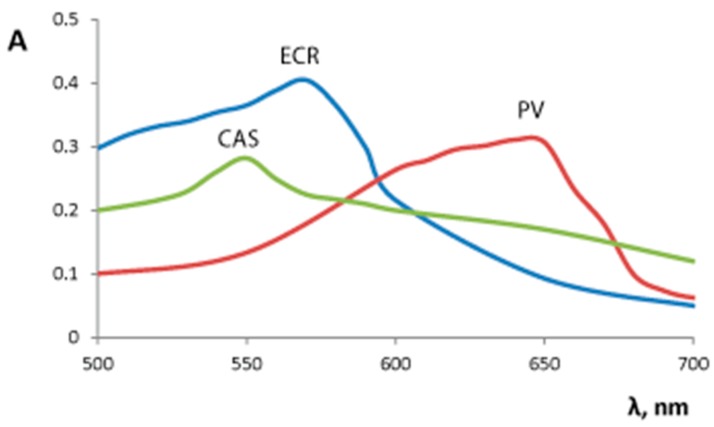
Absorption spectra of silica–titania xerogels after the interaction with 1 × 10^−4^ M triphenylmethane dyes solution. PV—pyrocatechol violet (pH 7.3), ECR—eriochrome cyanine R (pH 2.0), CAS—chrome azurol S (pH 3.0). m/V = 0.10 g/5.0 mL, time of contact is 20 min.

We studied the influence of Reaction (1) conditions (pH, time of contact, λ) on the xerogel absorbance. The contact of the silica–titania xerogels with TPMD resulted in the xerogels color change from white to violet (ECR, CAS) or blue (PV). The UV–vis absorption spectra of colored xerogels, obtained by these reactions, are given in Figure 1. Xerogel absorption maxima were observed at 570 nm for silica–titania xerogel interaction with ECR, at 640 nm for silica–titania xerogel interaction with PV, and at 550 nm for silica–titania xerogel interaction with CAS (Table 1). The difference may be explained by different structure of these TPMD: ECR and CAS, unlike PV, contain not only hydroxylic and sulfonic groups, but also carboxylic groups [26]. Maximal absorbance value at the absorption maximum was observed for silica–titania xerogel interaction with ECR. For each TPMD xerogel absorbance measurement was read at the corresponding absorption maximum.

**Figure 2:**
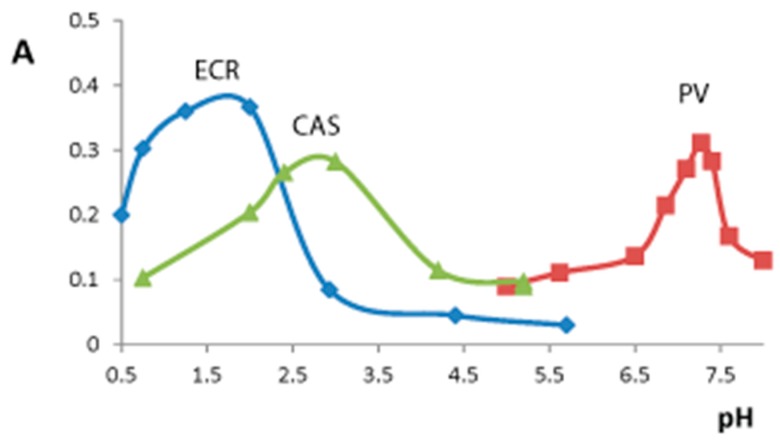
The effect of pH on silica–titania xerogels absorbance after the interaction with 1 × 10^−4^ M triphenylmethane dyes solution. PV—pyrocatechol violet (λ 640 nm), ECR—eriochrome cyanine R (λ 570 nm), CAS—chrome azurol S (λ 550 nm). m/V = 0.10 g/5.0 mL, time of contact is 20 min.

In present work the influence of pH on the TPMD-titanium(IV) complex formation was studied in the range of 1.0–8.0 (Figure 2). Maximal xerogel absorbance values were observed at pH 2.0 for silica–titania xerogel interaction with ECR, at pH 7.3 for silica–titania xerogel interaction with PV, and at pH 3.0 for silica–titania xerogel interaction with CAS (Table 1). These conditions were used in the following experiments. Such difference in pH optima values can be attributed to different pKa values of the studied TPMD; for all the studied dyes three of four acid groups stay protonated at the chosen pH values [31,32,33], which allows describing them as HL in Equation (1).

**Figure 3:**
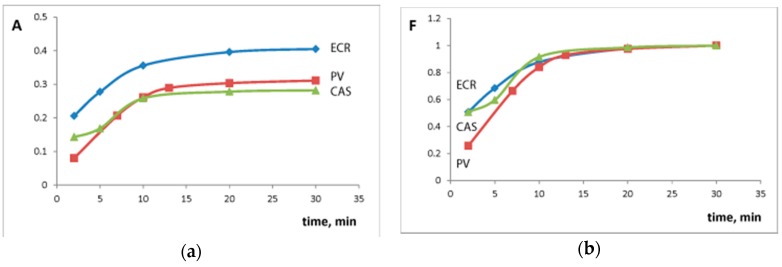
The kinetics of the interaction of silica–titania xerogels with 1 × 10^−4^ M triphenylmethane dyes solution: (**a**) dependence of xerogel absorbance on time; (**b**) dependence of conversion on time. PV—pyrocatechol violet (λ 640 nm, pH 7.3); ECR—eriochrome cyanine R (λ 570 nm, pH 2.0); CAS—chrome azurol S (λ 550 nm, pH 3.0). m/V = 0.10 g/5.0 mL.

Textural characteristics of silica–titania xerogel influence the heterogeneous reaction kinetics. Silica–titania xerogel was characterized by large surface area and high porosity which allows fast interaction with TPMD (Table 2, Si-Ti). For this material and all the studied TPMD equilibrium was reached at 10 min after the reaction start (Figure 3a). An earlier developed approach [34] was used to calculate half-reaction periods (T_1/2_) (Table 1). The degree of attaining equilibrium or conversion (F) was calculated as following: F(t) = A_t_/A_max_, where A_max_ is the absorbance of the xerogel at 30 min and A_t_ is the absorbance of the xerogel at given time (Figure 3b). The function −ln(1 − F(t)) is a linear function of time [34]. Half-reaction period (time when F = 0.5) can be found using these linear equations. All the interactions were found to be rather fast (T_1/2_ < 5 min). Silica–titania xerogel interaction with ECR was characterized with the smallest T_1/2_ value (2.1 min).

To further evaluate the interaction of silica–titania xerogels with TPMD, we determined the capacity values using adsorption isotherms method. We prepared a set of TPMD solutions with different concentrations (C_initial_, M) in the 2.0 × 10^−5^ M–1.0 × 10^−3^ M range and measured the solutions absorbance after the interaction with silica–titania xerogel, which allowed calculating the residual TPMD concentration (C_residual_, M) in solution. The difference ΔC = C_initial_ − C_residual_ showed the amount of TPMD adsorbed on silica–titania xerogel. The ΔC value increased with C_initial_ increase until it became constant (ΔC_max_, M). Capacity values (mol/g) were calculated ΔC_max_∙m/V, where m is the weight of the xerogel (0.10 g) and V is the volume of the solution (5.0 mL) (Table 1). The greatest capacity was demonstrated for ECR (0.9 ± 0.2 µmol/g, *n* = 3, *P* = 0.95).

The complex stoichiometry (n) and the equilibrium constants (K_eq_) were determined with the help of the equilibrium shift method [28]. The equilibrium constant of the reaction (1) is defined in the Equation (2). The logarithmic form of this Equation (3) turns this equation into a linear one.

(2) $$K_{\mathrm{eq}}\;=\;[\equiv \bar{\mathrm{Ti}-L_{n}}]/([\equiv \bar{\mathrm{Ti}-{\left(\mathrm{OH}\right)}_{n}}{][\;\mathrm{HL}]}^{n}),$$

(3) $$\mathrm{lg}([\equiv \bar{\mathrm{Ti}-L_{n}}]/[\equiv \bar{\mathrm{Ti}-{\left(\mathrm{OH}\right)}_{n}}])=\mathrm{lg}K_{\mathrm{eq}}\;+\;\mathrm{nlg}[\;\mathrm{HL}].$$

**Figure 4:**
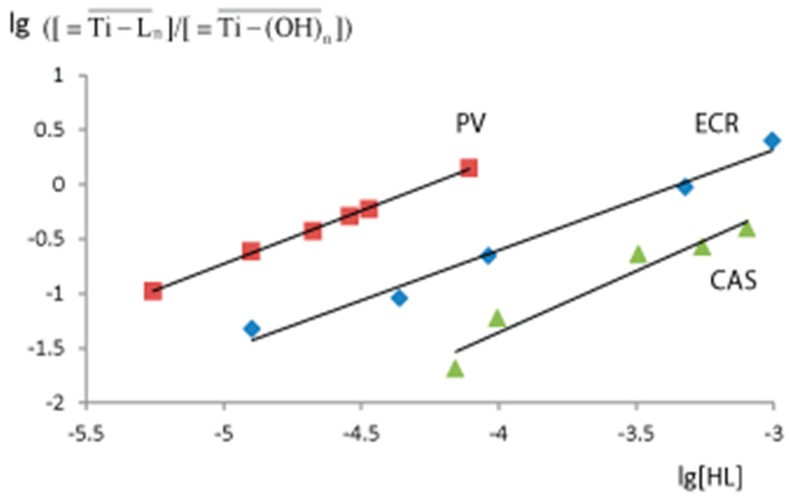
The dependence of lg$\left([\equiv \bar{\mathrm{Ti}-L_{n}}]/[\equiv \bar{\mathrm{Ti}-{\left(\mathrm{OH}\right)}_{n}}]\right)$ on lg[HL], where HL is a triphenylmethane dye. PV—pyrocatechol violet; ECR—eriochrome cyanine R; CAS—chrome azurol S. PV—pyrocatechol violet (λ 640 nm, pH 7.3, 10 min); ECR—eriochrome cyanine R (λ 570 nm, pH 2.0); CAS—chrome azurol S (λ 550 nm, pH 3.0). m/V = 0.10 g/5.0 mL; time of contact is 10 min.

$\left([\equiv \bar{\mathrm{Ti}-L_{n}}]/[\equiv \bar{\mathrm{Ti}-{\left(\mathrm{OH}\right)}_{n}}]\right)$ is calculated as A_i_/(A_ex_ − A_i_), where A_i_ is the xerogel absorbance after the contact with TPMD and A_ex_ is the xerogel’s absorbance after the contact with the excess of TPMD. [HL] is the concentration of TPMD in solution after this reaction, measured by its own absorbance. lg$\left([\equiv \bar{\mathrm{Ti}-L_{n}}]/[\equiv \bar{\mathrm{Ti}-{\left(\mathrm{OH}\right)}_{n}}]\right)$ is a linear function of lg[HL] (3), so the parameters of this function (n and K_eq_) can be calculated using simple linear regression analysis of the experimental data (Figure 4). The complex stoichiometry was 1:1 for all the studied TPMD and the equilibrium constants are given in Table 1. The smallest K_eq_ (1200 M^−1^) was observed for ECR.

Among three studied TPMD complexes with silica–titania xerogel, incorporated titanium(IV)-ECR complex was chosen for the construction of a sensor material for oxalate determination. This complex is characterized by the shortest half-reaction period, by the smallest K_eq_ which could allow easier complex decomposition by oxalate, and the greatest capacity which could allow oxalate determination in a broad analytical range.

### 3.2. Analytical Application

Sensor material for oxalate determination was synthesized by drying the colored silica–titania xerogel after its interaction with 5.0 × 10^−4^ M ECR (pH 2.0, 30 min); this ECR concentration was chosen because the maximum capacity was reached for ECR concentrations equal or greater than 5.0 × 10^−4^ M. Obtained sensor material (Si-Ti/ECR) retained its color (absorption maximum remained 570 nm) during storage for at least one year. 

Surface area and porosity BET analysis and BJH pore distribution analysis of Si-Ti/ECR showed changes in the textural characteristics compared to unmodified silica–titania xerogel (Table 2). The decrease in surface area and pore volume can be explained by the ECR adsorption on the xerogel surface. All the parameters except average pore diameter decreased by ~20% and the average pore diameter did not change; both these facts suggest that ECR is adsorbed evenly on the xerogel surface.

**Figure 5:**
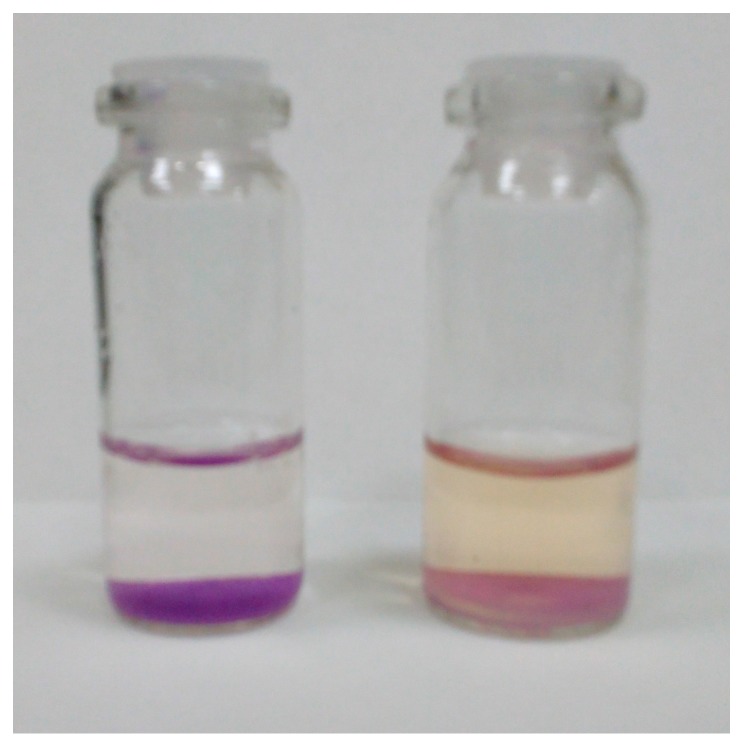
The color of Si-Ti/ECR before (left) and after (right) interaction with 200 mg/L oxalate solution (pH 3.0 m/V = 0.10 g/ 5.0 mL, time of contact is 15 min).

The interaction of Si-Ti/ECR with oxalate solution led to partial discoloration of the material, while the solution gained light yellow color (Figure 5). These changes can be explained by oxalate causing the decomposition of colored Ti(IV)-ECR complex and the following diffusion of ECR into the solution. This process can be described with the equation

(4) $$\equiv \bar{\mathrm{Ti}-L_{n}}+mOx^{2-}\;\to \;\equiv \bar{\mathrm{Ti}-L_{n-m}{\mathrm{Ox}}_{m}}+\mathrm{mL}.$$

**Figure 6:**
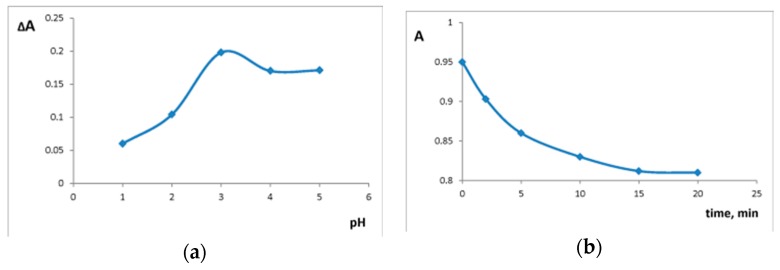
(**a**) The effect of pH on ΔA = A_0_ − A_200_, where A_0_ is Si-Ti/ECR absorbance in absence of oxalate and A_200_ is Si-Ti/ECR absorbance in presence of 200 mg/L oxalate (λ 570 nm, time of contact is 15 min); (**b**) kinetics of the interaction of Si-Ti/ECR with 200 mg/L oxalate solution (λ 570 nm, pH 3.0). m/V = 0.10 g/ 5.0 mL.

Si-Ti/ECR absorbance maximum did not change after interaction with oxalate; Si-Ti/ECR absorbance at 570 nm was used as analytical signal. Medium acidity and reaction time influence on the interaction of oxalate and Si-Ti/ECR was studied. Sensor material absorbance was measured in the range of pH 1.0–5.0, both in the absence and presence of oxalate. The maximal difference in the absorbances was observed at pH 3.0 (Figure 6a). The equilibrium of the interaction between oxalate and Si-Ti/ECR was reached in 15 min (Figure 6b).

**Figure 7:**
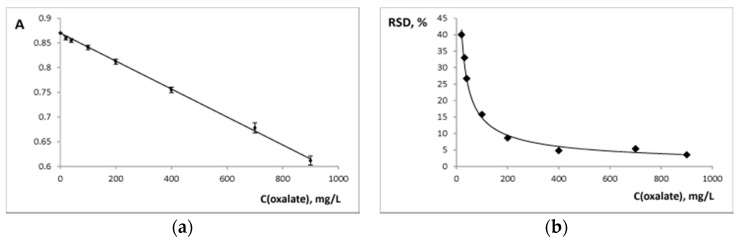
(**a**) Calibration curve for oxalate determination with Si-Ti/ECR (λ 570 nm, pH 3.0, m/V = 0.10 g/ 5.0 mL, time of contact is 15 min), standard deviations for absorbance are given (*n* = 3); (**b**) The dependence of relative standard deviation of concentration of the oxalate found (RSD) on the concentration of the oxalate added (*n* = 3).

Chosen conditions (pH 3.0, time of contact 15 min) were used to develop the analytical procedure of solid phase spectrophotometric determination of oxalate in food samples. Calibration curve was constructed using oxalate solutions in the 10–2000 mg/L range (m/V = 0.10 g/ 5.0 mL, m is the weight of the xerogel and V is the volume of the solution). A linear analytical curve (A = −2.8 × 10^−4^∙C + 0.87, R^2^ = 0.9982) was constructed (Figure 7a). The limit of detection (LOD) was calculated as 3∙standard deviation of the blank absorbance divided by the slope value and equaled 10.5 mg/L or 0.05 mg of oxalate (*n* = 7). The limit of quantification (LOQ) was calculated as 10∙standard deviation of the blank absorbance divided by the slope value and equaled 35 mg/L. Relative standard deviation of oxalate concentration calculated with calibration curve decreased with the increase of oxalate concentration (Figure 7b). The analytical range of the proposed procedure was 35–900 mg/L. We compared our procedure to another solid phase spectrophotometric procedure for oxalate determination in urine which is characterized by a LOD of 1.3 mg/L or 0.13 mg of oxalate (m/V = 0.10 g/100.0 mL) [22]. Our sensor material is characterized by better sensitivity.

Taking the LOQ value into account, proposed procedure allows determination of oxalate content above 35 mg/100 g if described sample treatment is used (10.0 g of sample is boiled in 100.0 mL of water). Vegetables and spices samples oxalate content is relatively high (10–1000 mg/100 g) and oxalate content in fruits, juices, and teas samples is generally lower (0.5–10 mg/100 g) [7]. Suggested procedure can be applied to oxalate determination in vegetables and spices with high oxalate content; a different sample treatment is required for oxalate determination in other food products.

The interference of the substances commonly present in plant samples, inorganic anions (chloride, phosphate, nitrate, sulfate), organic acids (citric, ascorbic, tartaric, gallic), and sucrose was studied. Interference thresholds for interfering substances are given in Table 3 and compared to the thresholds for other spectroscopic methods described in literature; for all inorganic anions, interference thresholds were the greatest for our procedure. In our experiments, ascorbic acid did not interfere the determination in the concentrations as high as 200 mg/L. This is especially important for food analysis because ascorbic acid is present in most plants. Other methods described in literature can only be used at lower concentrations of ascorbic acid (0.2 mg/L [10], 176 mg/L [12], 6.0 mg/L [19]). We studied the interference of phenolic compounds using gallic acid because total phenol content in plants is commonly measured in gallic acid equivalents (GAE). The interference threshold was found to be 60 mg/L of gallic acid. This threshold allows oxalate determination in many vegetables, spices, and herbs; for example, samples analyzed in the present work contain 20–200 mg GAE /100 g of fresh weight [35,36,37,38] or 4–40 mg/L GAE in the treated samples. In the present work, xerogel absorbance was measured at 570 nm, which reduced the influence of phenolic compounds that form colored complexes with titanium(IV) with the absorbance maxima at 400–450 nm [29].

The proposed procedure was applied to oxalate determination in food samples. The results of the recovery test for solid phase spectrophotometric determination of oxalate in food samples are given in Table 4. Results of the recovery test demonstrated the reliability of the proposed procedure. Oxalate content in five food samples was determined using standard addition method (Table 5), the results are in good agreement with the oxalate content data that can be found in literature [2,7] and with HPLC results.

## 4. Conclusions

In this work we studied the interaction between silica–titania xerogel and triphenylmethane dyes (pyrocatechol violet, chrome azurol S, eriochrome cyanine R). The complex forming reaction between xerogel incorporated titanium(IV) and eriochrome cyanine R was characterized by the shortest half-reaction period and the smallest equilibrium constant. Silica–titania xerogel sorption capacity was also calculated and the greatest value was also observed for eriochrome cyanine R. Eriochrome cyanine R was selected for the construction of a sensor material.

New sensor material (Si-Ti/ECR) was prepared and applied for solid phase spectrophotometric determination of food oxalates. The interaction of Si-Ti/ECR with oxalate ions has been described: the presence of oxalates causes Si-Ti/ECR discoloration; Si-Ti/ECR absorbance is used as analytical signal. The proposed procedure is characterized by broad analytical range of 35–900 mg/L and rather high thresholds for interfering anions, which allows the use of only very simple sample treatment steps. The time of analysis is 15 min, which is similar to the time of analysis for biosensors; however, Si-Ti/ECR is much more stable (one year compared to one month for most biosensors). Proposed solid phase spectrophotometric procedure was applied for the determination of oxalates in food samples (sorrel, spinach, parsley, ginger, and black pepper), the results were in good agreement with HPLC oxalate determination.

## Figures and Tables

**Table 1:** Characteristics of the interaction of silica–titania xerogels with triphenylmethane dyes.

Triphenylmethane Dye	λ_max_, nm	pH Optimum	T_1/2_, min	K_eq_, M^−1^	Capacity, µmol/g (*n* = 3, *P* = 0.95)
Pyrocatechol violet	640	7.3	4.2	13,500	0.5 ± 0.1
Eriochrome cyanine R	570	2.0	2.1	1200	0.9 ± 0.2
Chrome azurol S	550	3.0	2.5	1400	0.8 ± 0.2

**Table 2:** Comparison of textural characteristics of unmodified silica–titania xerogel (Si-Ti) and sensor material (Si-Ti/ECR).

	BET Surface Area, m^2^/g	Micropore Area, m^2^/g	Total Pore Volume, cm^3^/g	Micropore Volume, cm^3^/g	Average Pore Diameter, Å
Si-Ti	617	250	0.30	0.11	19.2
Si-Ti/ECR	520	194	0.25	0.09	19.3

**Table 3:** Interference thresholds for different spectroscopic methods for oxalate determination.

Interfering Compound	Interference Threshold, mg/L	Reference
Chloride	3.5 × 10^3^	[10]
	3.0 × 10^1^	[21]
	1.8 × 10^4^	[22]
	7.1 × 10^1^	[23]
	4.0 × 10^3^	Present work
Phosphate	5.0	[20]
	6.0 × 10^−2^	[21]
	4.8 × 10^2^	[22]
	1.2 × 10^4^	Present work
Nitrate	2.8 × 10^2^	[20]
	6.0 × 10^1^	[21]
	9.3 × 10^3^	[22]
	6.2 × 10^1^	[23]
	1.1 × 10^4^	Present work
Sulfate	2.0 × 10^1^	[20]
	6.0	[21]
	4.8 × 10^2^ (in presence of BaCl_2_)	[22]
	7.6 × 10^1^	[23]
	2.0 × 10^3^	Present work
Citric acid	4.8 × 10^3^	[10]
	4.0	[20]
	6.0 × 10^−2^	[21]
	1.0 × 10^4^	Present work
Ascorbic acid	0.2	[10]
	6.0	[21]
	2.0 × 10^2^	Present work
Tartaric acid	7.5 × 10^4^	[10]
	3.0 × 10^−2^	[21]
	2.0 × 10^2^	Present work
Gallic acid	0.6 × 10^2^	Present work
Sucrose	8.6 × 10^4^	[10]
	2.0 × 10^3^	Present work

**Table 4:** Recovery test of solid phase spectrophotometric determination of oxalate in food samples (*n* = 3).

Sample	Oxalate Added, mg/L	Recovery, %
Sorrel	184	93.2
	368	107.7
Spinach	184	96.7
	368	98.7
Black pepper	184	83.6
	368	103.9
Parsley	184	100.3
	368	105.6
Ginger	184	93.0
	368	95.5

**Table 5:** Solid spectrophotometric (Si-Ti/ECR) and HPLC determination of oxalate in food samples (*n* = 3, *P* = 0.95).

Sample	Oxalate Content, mg/100 g (RSD, %)	
	Proposed method	HPLC
Sorrel	1079 ± 158 (8.5)	995 ± 24 (1.4)
Spinach	811 ± 152 (10.9)	720 ± 15 (1.2)
Black pepper	876 ± 210 (13.9)	857 ± 18 (1.2)
Parsley	782 ± 80 (5.9)	835 ± 17 (1.4)
Ginger	662 ± 150 (13.2)	634 ± 16 (1.5)
